# A Novel Compound Heterozygous Mutation in the DNAH11 Gene Found in Neonatal Twins With Primary Ciliary Dyskinesis

**DOI:** 10.3389/fgene.2022.814511

**Published:** 2022-02-28

**Authors:** Shumei Dong, Fei Bei, Tingting Yu, Luming Sun, Xiafang Chen, Hui Yan

**Affiliations:** ^1^ Department of Neonatology, Shanghai Children’s Medical Center, School of Medicine, Shanghai Jiaotong University, Shanghai, China; ^2^ Department of Medical Genetics, Shanghai Children’s Medical Center, School of Medicine, Shanghai Jiaotong University, Shanghai, China; ^3^ Pediatric Translational Medicine Institute, Shanghai Children’s Medical Center, School of Medicine, Shanghai Jiao Tong University, Shanghai, China; ^4^ Department of Fetal Medicine and Prenatal Diagnosis Center, Shanghai First Maternity and Infant Hospital, Tongji University School of Medicine, Shanghai, China

**Keywords:** monozygotic twins, situs inversus, *DNAH11*, primary ciliary dyskinesia, whole-exome sequencing

## Abstract

**Background:** Primary ciliary dyskinesia (PCD) is a rare genetically heterogeneous disorder of motile cilia. Common features of PCD include upper and lower respiratory tract disease, secretory otitis media, situs inversus and fertility problems. To date, although several PCD-associated genes have been identified, the genetic causes of most PCD cases remain elusive.

**Methods:** In this case study, we analyzed the clinical and genetic data of one case of monochorionic diamniotic twins which were suspected of having PCD on the basis of clinical and radiological features including situs inversus, recurrent wet cough and sinusitis as well as varying degrees of respiratory distress. Whole-exome sequencing was performed to identify variants of the *DNAH11* gene in the twins. Sanger sequencing and real-time quantitative polymerase chain reaction (RT-qPCR) were used for validation of *DNAH11* variants both in the patient and the twins.

**Results:** In the twins, we found a novel mutation at c.2436C > G (p.Y812 *) and a pathogenic deletion encompassing 2.0 Kb of 7P15.3 ([GRCh38] chr7: g.21,816,397-21,818,402). The deleted region included exons 64 and 65 of *DNAH11*. Sanger sequencing also revealed that the twins’ father was a carrier of heterozygous C.2436C > G and a heterozygous deletion was detected in the mother. No other clinically relevant genetic variants were identified.

**Conclusion:** We describe a novel *DNAH11* gene compound heterozygous mutation in newborn twins with PCD and recommend that PCD diagnosis should be considered in newborns presenting with respiratory distress and/or situs inversus. Early diagnosis and treatment of PCD will help control disease progression and improve the patient’s quality of life.

## 1 Introduction

Primary ciliary dyskinesia (PCD, MIM #244400) is a rare autosomal recessive, genetic disorder of motile cilia. The estimated prevalence of PCD is between 1/2,000 to 1/40,000 live births (Goutaki et al., 2016). PCD has been associated with abnormal ciliary ultrastructure and function leading to the failure of mucus clearance. PCD manifests as upper and lower respiratory tract abnormalities, secretory otitis media, situs inversus, and fertility issues (Rubbo and Lucas, 2017). Recurrent otitis media and airway infection are commonly observed during the neonatal period (Mirra et al., 2017). Unless a dextrocardia is detected on a chest X-ray, PCD diagnosis is usually delayed. At present, the median age at PCD diagnosis in the Chinese pediatric population is 7 years (range: 2 months to 14 years), which is much later compared with pediatric patients in Europe and North American (Guan et al., 2021).

In this study, we described a monochorionic -diamniotic twin pregnancy in which one fetus was diagnosed with situs inversus during routine prenatal ultrasonography. This led to the genetic testing of both the parents and the twins. We found that the twins were carriers of a pathogenic compound heterozygous mutation in the *DNAH11* gene. The twins diagnosed with PCD presented respiratory symptoms shortly after birth. This study may contribute to the understanding of PCD among neonatologists and pediatricians, and provide information for obstetricians and genetic counselors to guide prenatal and postnatal care.

## 2 Materials and Methods

### 2.1 Patients and Clinical Evaluation

A 33-year-old Chinese woman who conceived naturally, gravida 1, para 0, presented for routine fetal anatomy ultrasound at 21 weeks gestation. Upon examination, a monochorionic diamniotic twin pregnancy was diagnosed by ultrasonograpy. A thin intertwin dividing membrane and single placenta complicated with selective growth restriction type III (type III sIUGR) and inter-twin amniotic fluid discordance (AFD) were observed. The larger twin showed signs of situs inversus and polyhydramnios. Growth diameter line below two standard deviations and intermittent absence of end-diastolic umbilical artery flow were observed in the smaller twin. Decompression amniocentesis was performed for the discordant twin at 21 weeks and 4 days gestation. Because of the association between situs inversus and PCD, our medical institution offered genetic counseling, which included an explanation on exome sequencing. Informed consent was obtained before the procedure.

Obstetric ultrasound was performed regularly throughout the pregnancy in the antenatal care ward. Cesarean section was performed due to uncontrolled uterine contractions associated with polyhydramnios. Two preterm monochorionic diamniotic male twins were born at 32 weeks and 4 days gestation. Antenatal steroids were administered 2 weeks before delivery. The second twin (Twin B) was born 30 s later than his brother (Twin A). There were no clinical symptoms, laboratory, or histological signs of chorioamnionitis. The results of complete blood count, C-reactive protein (CRP), erythrocyte sedimentation rate (ESR), serum albumin, and serum iron levels all showed protective effects. The father was 33 years old. The parents were non-consanguineous according to self-report, without an unremarkable family genetic history.

### 2.2 Whole-Exome Sequencing and Bioinformatics Analysis

Considering that the twins were monochorionic identical twins and twin A presented with situs inversus, WES was first performed on twin A and his parents. Twin A’s DNA sample, obtained through amniocentesis and parents’ peripheral blood DNA were used for whole-exome sequencing to identify causal genetic variants. Briefly, 3 μg DNA was sheared to fragments of 150–200 bps in size. An adaptor-ligated library was prepared using the paired-end sequencing library prep kit (Agilent Technologies, Santa Clara, CA, United States). Both coding exons and flanking intronic regions were enriched using the Agilent SureSelect XT Human All Exon V6 reagent kit (Agilent Technologies, Santa Clara, CA, United States). Clusters were then generated by isothermal bridge amplification using the Illumina cBot station, and sequencing was performed using the Illumina HiSeq 2500 System (Illumina, San Diego, CA, United States). Burrows-Wheeler Alignment tool (BWA) v0.2.10 was used for sequence alignment to the Human Reference Genome (NCBI build 37, hg 19). Data quality was assessed using FastQC (version 0.11.2). The read data were uploaded to the Ingenuity Variant Analysis platform (Qiagen, United States) for mutation screening and interpretation. Copy number detection and visualization of WES were performed using the bioinformatics tool CNVkit.2.3.

### 2.3 Sanger Sequencing and Real-Time Quantitative Polymerase Chain Reaction

Sanger sequencing of *DNAH11* was performed in the twins A, B and their parents. Exon 14 of the *DNAH11* was amplified by PCR from the genomic DNA of the twins A, B and their parents (primer sequence available on request). Direct DNA sequencing using an ABI3730XL sequencer (Applied Biosystems, Foster City, CA, United States) was applied for sequence analysis of the PCR products.

To confirm the deleted region in *DNAH11* observed from WES data, we performed RT-qPCR using SYBR Green PCR master mix (Ambion; Thermo Fisher Scientific) and genomic DNA from the twins A, B and their parents. The primers used for amplification of *DNAH11* were as follows: *DNAH11* exon 64 forward, 5′- ATGTCCACCGAAAATGCC -3′ and reverse, 5′- AAA​AAT​CAG​ACC​CAC​TTC​ACA​G -3′. Beta-Actin gene was used as the internal control. All PCR assays were performed in triplicate. Fluorescence intensity, as an indicator of amplicon concentration, was calculated based on the equation ΔRn = (Rn^+^)–(Rn^−^) (reporter signal fluorescence minus normalized background). The signal amplification (ΔRn) was then plotted against PCR cycles to generate cycle threshold number (Ct) values. Fluorescence signals were analyzed using an automatic setting of the threshold line in the StepOne™ software (v.2.3). The Ct value was the initial cycle number at which amplification was detected as exceeding an arbitrary threshold. The specificity of the amplified products was evaluated by melting curve analysis. Relative copy number changes were calculated using the 2^−ΔΔCT^ method.

## 3 Results

### 3.1 Clinical Features

#### 3.1.1 Twin A

Twin A birth weight was 1,800 g (10th∼50th percentile for gestational age) with Apgar scores of 8 and 8 at 1 and 5 min, respectively. The umbilical arterial pH and hemoglobin levels were 7.255 and 16.9 g/dl, respectively. The infant developed respiratory distress after delivery. He was immediately intubated and given one dose of surfactant (120 mg Surfactant Curosurf suspension), before being transferred to the Neonatal Intensive Care Unit (NICU) for further management. On admission to the NICU, twin A underwent invasive ventilation for 3 days, followed by 10 days of weaning with non-invasive ventilation. Afterwards, twin A was released from ventilator support.

**FIGURE 1:**
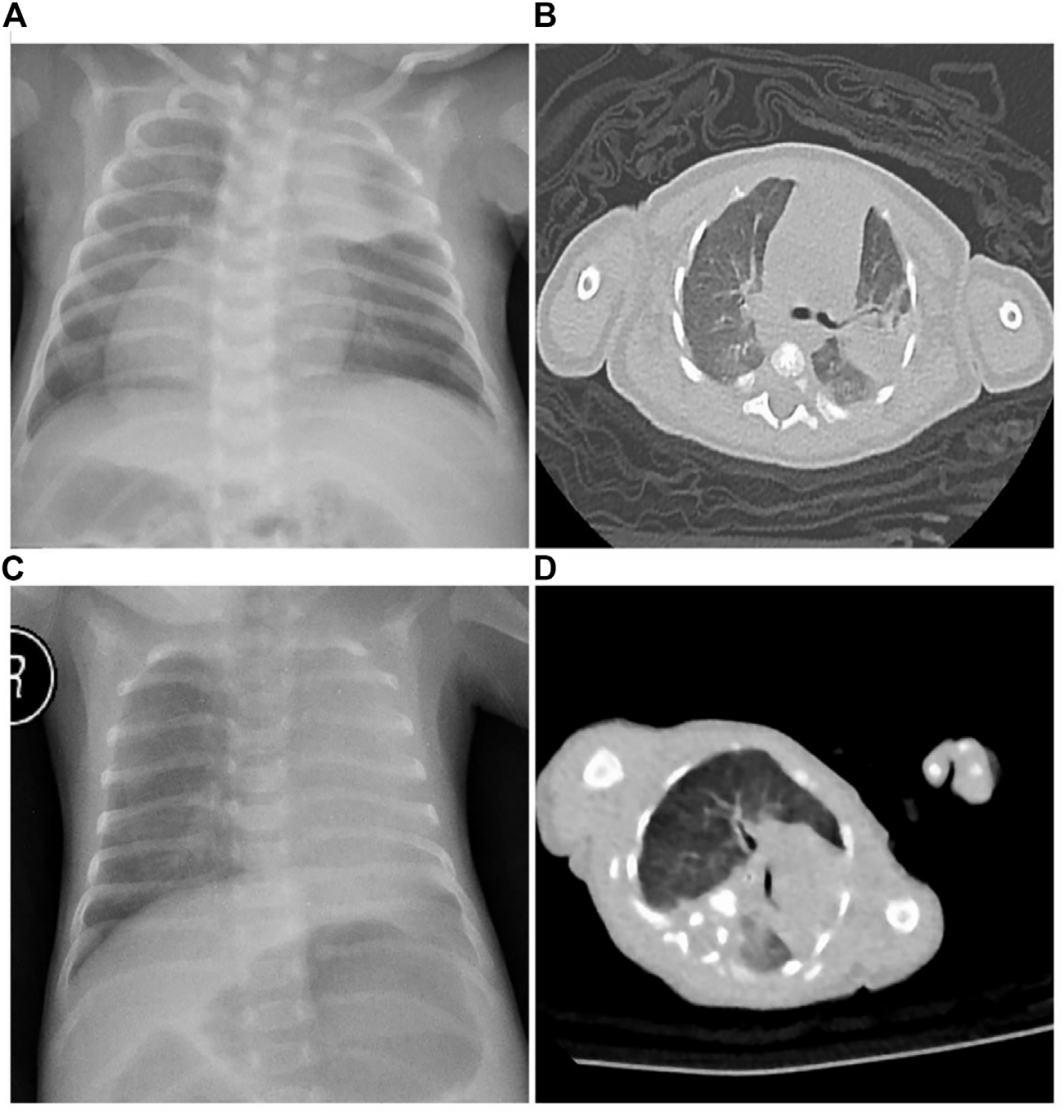
Radiological examination of the twins. **(A)** Twin A chest X-ray showed consolidation and atelectasis in the left upper lung, heart inversus and right-sided placement of the stomach bubble. **(B)** Twin A chest CT revealed bilateral lung infection with consolidation in the left upper lobe and mirror-image dextrocardia. **(C)** Twin B chest X-ray showed atelectasis in the left upper lung and normal cardiac and stomach placement. **(D)** Twin B chest CT revealed atelectasis in the left upper lung.

On day 34 after birth, Twin A was transferred to our hospital (Shanghai Children’s Medical Center affiliated to Shanghai Jiaotong University School of Medicine, China) with a 1-day history of respiratory distress, wet cough, and yellow-green phlegm. The child had no fever, wheezing, or cyanosis. On physical examination, body length: 48 cm (P50∼90), weight: 2,800 g (P50∼90), and head circumference: 33 cm (P50∼90). His vital signs on admission were: respiratory rate of 48 per min, heart rate of 126 per min, blood pressure of 69/48 mmHg, and body temperature of 36.4°C. A small amount of yellow secretion was observed in his nasal cavity. Wet lung rales were observed on both sides. Heart sounds were more pronounced along the right sternal border. Examination results of the abdomen and other areas were normal. Complete blood test results were: Hb 8.6 mg/dl, WBC 19.04 × 109/L, N% 45.8%, platelets 355 × 109/L, and CRP 1.2 mg/dl. Sputum culture revealed *Klebsiella pneumoniae*. Chest radiograph showed consolidation and atelectasis in the left upper lung and heart inversus (Figure 1A). Chest computed tomography also revealed bilateral lung infection with consolidation in the left upper lobe and mirror-image dextrocardia (Figure 1B). Cardiac ultrasound showed situs inversus with dextrocardia and atrial septal defect (.25 cm, bidirectional shunt). Abdominal ultrasound suggested right-sided stomach and spleen, left-sided liver, and IVC to the left of the aorta.

During hospital stay, twin A was given Sulperazone, oxygen hood, sputum suction, physical therapy, and other supportive treatments. After 12 days of treatment, his respiratory symptoms were improved, then he was discharged. A follow-up at 10 months after birth showed recurrent wet cough and sinusitis. Twin A was required to undergo nutritional support, avoid cross infections, home atomization, and regular follow-up in the Respiratory Department.

#### 3.1.2 Twin B

Birth weight was 1,275 g (3rd∼10th percentile for gestational age; 29% growth discordance) with Apgar scores of 8 and 9 at 1 and 5 min, respectively. Umbilical arterial pH and hemoglobin levels were 7.252 and 22.1 g/dl, respectively. No morphological anomalies were observed. The neonatal course was notable for mild respiratory distress in the delivery room, which required continuous positive airway pressure and transfer to the neonatal intensive care unit. On admission to the NICU, Twin B was ventilated nasally by continuous positive airway pressure (nCPAP). On physical examination, Twin B’s anthropometric measurements were as follows: weight 1,275 g (3∼10 percentile), length 40 cm (3∼10 percentile), and head circumference 27 cm (3–10 percentile). His vital signs on admission were: respiratory rate of 47 per min, heart rate of 135 per min, blood pressure of 55/44 mmHg, and body temperature of 36.6°C. A small amount of yellow secretions were observed in his nasal cavity. Breathing was stable under the support of non-invasive ventilation. The presence of wet lung rales was observed on both sides. Complete blood test results were: Hb 187 mg/dl, WBC 4.58 × 109/L, N% 25.5%, platelets 119 × 109/L, and CRP < .8 mg/dl. Sputum culture revealed *Klebsiella pneumoniae*. Chest X-ray showed atelectasis in the left upper lung (Figure 1C). Chest computed tomography revealed bilateral lung infection, mediastinal pulmonary hernia, and 11 pairs of ribs (Figure 1D). Cardiac ultrasound showed an atrial septal defect (.38 cm, bidirectional shunt). No obvious abnormality was found in other examinations. A fiberoptic bronchoscopy examination with a bronchoalveolar lavage suggested inflammation of the endotracheal and bronchial membranes. In addition, numerous secretions were observed obstructing the upper left lobe. Alveolar lavage fluid culture was negative.

During hospitalization, twin B was given nutritional support, anti-infection medication, non-invasive respiratory support, sputum suction, physical therapy, and other supportive treatments. After 46 days of treatment, feeding tolerance and respiration were improved. Follow-up symptoms and recommendations were similar to Twin A.

### 3.2 Identification of Novel *DNAH11* Compound Heterozygous Mutations

WES results of the twin A and his parents indicated that twin A had novel compound heterozygous mutations in the *DNAH11* gene. We detected a heterozygous nonsense mutation (c.2436C > G, p.Y812 *) in exon 14 of the *DNAH11* in the twin A and his father by WES, categorized as pathogenic according to ACMG guidelines (Richards et al., 2015). A deletion encompassing 2.0 Kb of 7P15.3 ([GRCh38] chr7:g.218,163,97-218,184,02) was also detected by WES from the twin A and his mother. The deleted region involved exons 64 and 65 of the *DNAH11* gene and was categorized as pathogenic based on ACMG guidelines (Richards et al., 2015) (the NGS analysis with coverage details and Insilico-analysis can be found in Supplementary Material).

**FIGURE 2:**
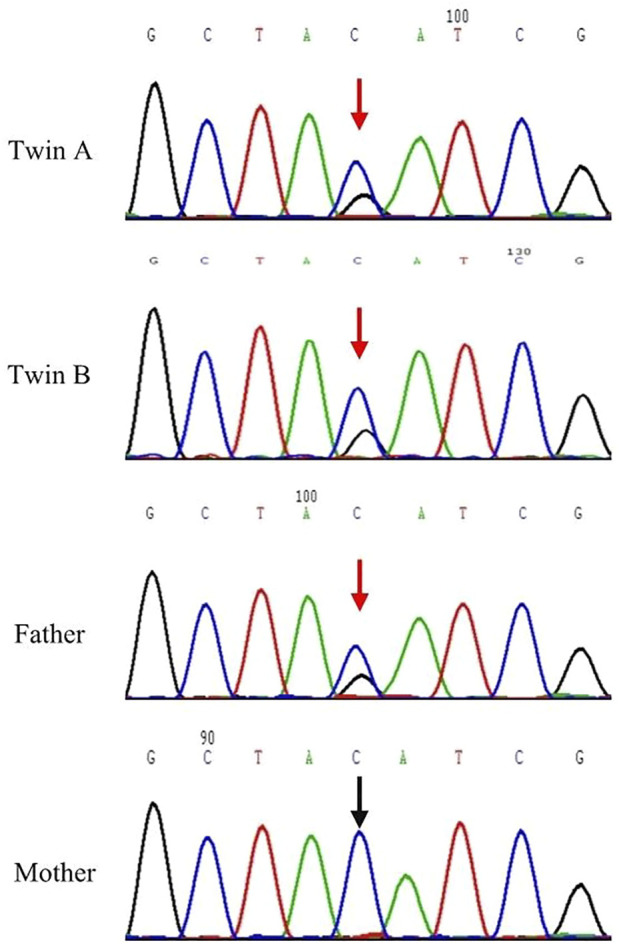
Sanger sequencing results for *DNAH11* c.2436 of Twins A, B and their parents. The *DNAH11* c.2436 C *>* G; p.Y812 * variant was detected in the Twins A, B and their father. Red arrows designate variant bases.

Sanger sequencing confirmed the nonsense mutation in the twins A, B and their parents. Sanger sequencing demonstrated that the twins A, B and their father had a heterozygous nonsense mutation within exon 14 (c.2436C > G, p.Y812 *) in the *DNAH11* gene, and their mother carried wild type gene in this location (Figure 2).

**FIGURE 3:**
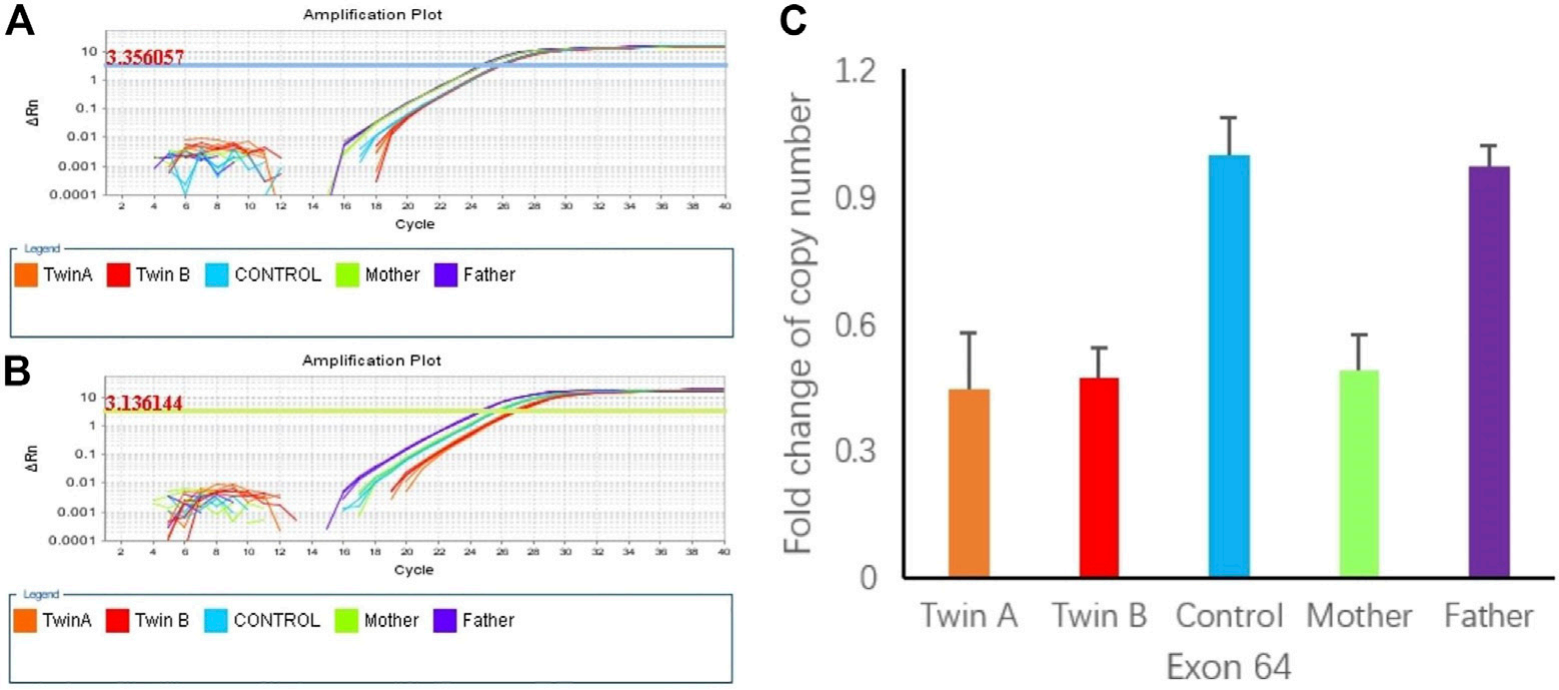
**(A)** Amplification plot of β-actin (3.350657 represent the flourescence thresholds value of beta-actin). **(B)** Amplification plot of DNAH11 exon 64 (3.1366144 represent the flourescence thresolds value of target gene). **(C)** Copy numbers of exon 64 of DNAH11 confirmed by qPCR using genomic DNA from the twins and the parents.

**FIGURE 4:**
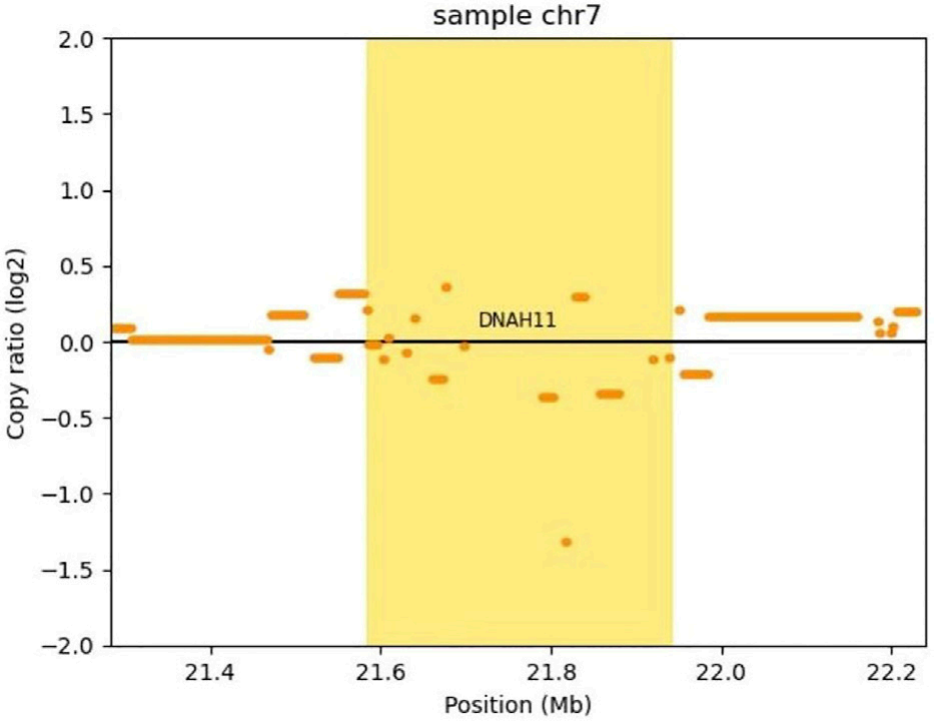
Copy number analysis of the DNAH11 gene deletion by CNVkit. CNVkit, a command-line toolkit for copy number analysis, was applied in our CNV analysis (version 0.9.9). It is designed for use with hybrid capture, including both whole-exome and custom target panels, and short-read sequencing platforms such as Illumina and Ion torrent. We built a reference from normal samples according to our exome’s capture positions, detected a CNV with log2 (copy ratio) <−1 in DNAH11 gene (bright yellow block), which means one copy loss.

To confirm the heterozygous deletion at chromosome 7P15.3, we performed real-time qPCR using genomic DNA from the twins and their parents. Figures 3A,B show the qPCR amplification curves of *DNAH11* gene exon 64 for twins A, B and their parents using beta-actin gene as internal reference. In Figure 3, 3.136144 and 3.356057 represent the fluorescence thresholds value of target gene and reference gene (beta-Actin) respectively. The qPCR results showed that 50% of the exon 64 copies were observed in the twins and their mother relative to the father (Figure 3C). Bioinformatics analysis further demonstrated the DNAH11 gene deletion (Figure 4).

## 4 Discussion

PCD mainly causes lesions in the ear, nose, lungs, fallopian tubes, and vas deferens. The clinical manifestations of PCD during childhood include chronic rhinosinusitis, secretory otitis media, recurrent respiratory tract infections, and bronchiectasis. The incidence of chronic rhinosinusitis in PCD patients is as high as 91.7%, while secretory otitis media is around 50% (Yue et al., 2019; Guo et al., 2020). Unexplained neonatal pneumonia, atelectasis, and respiratory distress in term and near-term newborns, as well as situs viscerum inversus may be present during the neonatal period (Lucas et al., 2020). In our study, the affected twins showed respiratory symptoms soon after birth, while imaging findings suggested pneumonia and atelectasis. After discharge, recurrent wet cough and yellow mucus were still observed during follow-up. PCD patients are usually diagnosed several years after the onset of symptoms. The age at diagnosis in the Chinese pediatric population ranges from 2 months to 14 years (Guan et al., 2021). Due to vast differences in the severity of respiratory symptoms in PCD patients, misdiagnosis is common in patients who do not manifest situs viscerum inversus.

PCD has been associated with situs inversus in 50% of the patients. Even in the same family, there may be cases of normal situs and situs inversus. This may be related to the effect of gene mutations that regulate the function of nodal cilia in the embryo (Struben et al., 2005). Animal studies have shown that the mouse *Dnahc11* gene (a mouse homolog of the *DNAH11* gene), encodes the heavy chain of the outer dynein arm of cilia, which is responsible for the movement of cilia, regulating the direction of fluid flow during embryonic development, and determining the location and distribution of viscera (Kawasumi et al., 2011; Lucas et al., 2014). Allelic mutations of the *Dnahc11* gene in mice cause immobility of the cilia and inversion of left-right body axis, resulting in internal organs to orientate randomly (Supp et al., 1997; Okada et al., 1999). The twins in this study were genetically identical and developed the same clinical disease. The discordant situs laterality observed in both twins further supported that the *DNAH11* gene mutation could induce phenotypic heterogeneity.

To date, over 40 distinct genes have been reported to cause PCD. Among them, the DNAH11 gene mutation accounts for 14.3% of PCD occurred in China (Xia et al., 2021). The *DNAH11* gene, which encodes the axonemal heavy chain dynein 11, comprises of 82 exons and extends over 353 kb of genomic sequence on chromosome 7p15.3-21. As a molecular motor protein in the axial-filament structure system, the axonemal heavy chain dynein has ATPase activity and is responsible for converting the chemical energy stored in ATP into mechanical energy needed for sliding between microtubules (Burgess et al., 2003; Lin et al., 2014). *DNAH11* mutations can cause ciliary dysfunction, altering ciliary beating and patterns, resulting in poor mucociliary clearance. Two novel *DNAH11* variations were found in our twin cases. The novel deletion in exon 64–65 of the *DNAH11* gene covering the segment of chromosome 7p15.3 that resulted in a functional change in dynein. In addition, p.Y812* detected in the twins was a novel nonsense mutation in exon 14, located within the mutational hot spot of the *DNAH11* gene. To the best of our knowledge, both mutations identified in the current study had not been previously reported in the literature in PCD patients. The two gene variants were not assigned a RS number in the dbSNP database. Our discovery of the *DNAH11* mutation sites that showed a pathogenic phenotype might further expand the variant database for the *DNAH11* gene (The molecular modeling of DNAH11 Wild Type Protein can be found in Supplementary Materials).

At present, there is no single gold standard diagnostic test for PCD. Current diagnosis requires a combination of clinical symptoms and technically demanding investigations. These include nasal nitric oxide (nNO) measurements, high-speed video microscopy analysis (HVMA), transmission electron microscopy (TEM), immunofluorescence microscopy (IFM) analysis, and gene analysis (Shapiro et al., 2016). The ciliary ultrastructure in PCD patients with *DNAH11* gene mutations is normal. Knowles et al. (2012) found that biallelic mutations in *DNAH11* were relatively common (22%) in PCD patients without a defined ciliary ultrastructural defect. The ultrastructure of cilia using transmission electron microscopy was found to be normal in 5 of 8 PCD children. *DNAH11* mutations have a more prominent effect on cilia function. Using HVMA, it was observed that *DNAH11* mutations can cause stiff beats, reduce bending capacity, and hyperkinetic beat of cilia, which leads to ineffective clearance function (Pifferi et al., 2010). Guo et al. (2020) reported that 6 of the 8 PCD children had ciliary stiff beat and reduced bending ability. Ciliary beat frequency was found to be clearly reduced in seven cases (<11 Hz). Within the proximal ciliary region, electron tomography has demonstrated a deficiency of >25% in the proximal outer dynein arm volume in all patients with *DNAH11* mutations (Shoemark et al., 2018). IFM analysis showed that *DNAH5* localized throughout the cilium, while *DNAH11* and *DNAH9* distinctly localize to the proximal and distal regions of respiratory cilia, respectively. *DNAH9* can heterodimerize with *DNAH5* in the proximal ciliary region when *DNAH11* is either defective or absent. However, *DNAH9* cannot functionally replace the partial ODA defect in the proximal ciliary region and the characteristic hyperkinetic beating in *DNAH11*- mutant cilia (Dougherty et al., 2016). This helps us understand why *DNAH11* mutations result in normal ciliary ultrastructure with hyperkinetic ciliary beating and reduced beating amplitude. Hence, *DNAH11* mutations with clinical characteristics of PCD need to be systematically diagnosed by other methods besides TEM.

Currently, therapeutic strategies for PCD are not based on validated disease-specific recommendations. The main treatment measures include infection control and prevention, airway clearance, administration of drugs to promote cilia movement, improvement in immunity, and surgical treatment, if necessary (Daniels and Noone, 2015). Pregnancy screening, prenatal diagnosis, and genetic counseling play an important role in PCD screening and diagnosis, as well as risk counseling and testing for potential carriers (Metcalfe, 2012; Lucas et al., 2016). In this study, whole-exome sequencing was used to identify pathogenic genes present in the twins. Our genetic diagnosis provided an important reason for gene screening and genetic counseling for family members of the probands. Recently, new gene-editing technologies have provided opportunities to treat PCD patients by restoring gene function and normalizing cilia motility (Lai et al., 2016). If the disease could be diagnosed early, appropriate prevention and treatment measures could effectively delay the progress of bronchiectasis and lung function damage and improve prognosis (McManus et al., 2003).

## 5 Conclusion

In the present investigation, we found a c.2436C > G nonsense mutation in addition to a deletion in exon 64–65 of the *DNAH11* gene. Our findings of these novel mutation sites add to the list of pathogenic variants for the PCD gene. The twins in this study were identical in genotype. The discordant situs laterality in both individuals further validated that *DNAH11* gene mutations could cause phenotypic heterogeneity. At present, there are only a few clinical cases of PCD reported during the neonatal period, with most of the diagnoses reported in older children. It is critical that neonatologists, pediatricians, and obstetricians understand the clinical characteristics of PCD at various ages for early diagnosis and treatment. Nevertheless, additional cases are required for genotype/phenotype correlation analysis and identify new gene variants to understand the etiology of PCD. This will be important to effectively prevent and control the disease and improve patient quality of life.

## Data Availability

The datasets presented in this study can be found in online repositories. The name of the repository and accession number can be found below: SRA, NCBI; PRJNA801086.
