# Supplementary material for: A Novel Compound Heterozygous Mutation in the DNAH11 Gene Found in Neonatal Twins With Primary Ciliary Dyskinesis
Source: Front Genet. 2022 Feb 28;13:814511. doi: 10.3389/fgene.2022.814511 (PMC8919259; doi:10.3389/fgene.2022.814511)
Supplement: Supplementary file 1 [file DataSheet1.docx]

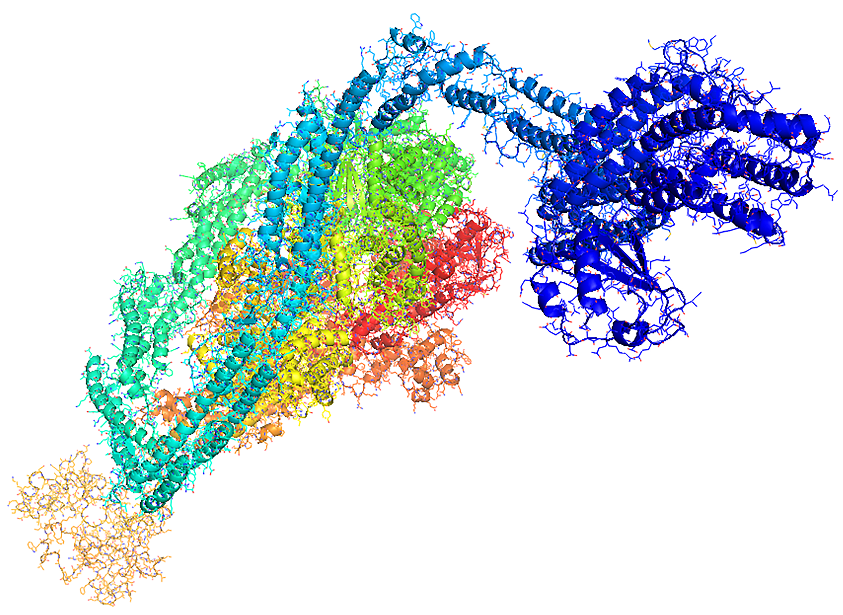


**A**


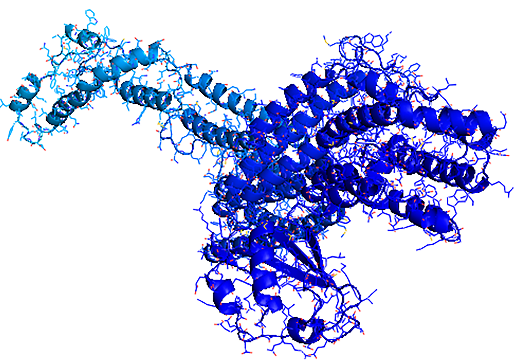


**B**

Fig 1. Structural modeling displayed conformational changes of the *DNAH11* p. (Y812 *) variant. **A** The cartoon representation of the

*DNAH11* protein. **B** The model of the mutant at residue 812 (p. Y812 *).

The protein structures of wild-type and variant-type were predicted via the online SWISSMODELtool (http://www.swissmodel.expasy.org), and the visualized structures were constructed using the PyMOL software (version 1.7, Schrödinger, LLC, Portland, U.S.A.).
